# Dietary vitamin a intake among Chinese adults: findings from CNTCS2015

**DOI:** 10.1186/s12937-018-0369-3

**Published:** 2018-06-11

**Authors:** Wenwen Du, Huijun Wang, Zhihong Wang, Jiguo Zhang, Chang Su, Xiaofang Jia, Ji Zhang, Hongru Jiang, Feifei Huang, Yifei Ouyang, Yun Wang, Li Li, Bing Zhang

**Affiliations:** National Institute for Nutrition and Health, Chinese Center for Disease Control and Prevention, 29 Nanwei Rd, Beijing, China

**Keywords:** Vitamin A, Retinol, Carotenes, China

## Abstract

**Background:**

Vitamin A plays an important role in human functions, which mainly come from foods. This study aims to examine dietary vitamin A intake and major food sources of Chinese adults.

**Methods:**

We analyzed the cross-sectional data from 12,246 adult aged 18 to 64 years old in 2015 China Nutritional Transition Cohort Study. Three consecutive 24-h dietary recalls combined with household weighing method were used to assess dietary vitamin A intake.

**Results:**

The average dietary vitamin A intakes were 480.9 μg retinol equivalents (RE) or 307.2 μg retinol activity equivalents (RAE). The carotenes and retinol intake of subjects were 2084.7 μg/day and 133.5 μg/day, respectively. Approximately 87% of adults consumed less vitamin A than the Chinese Estimated Average Requirement (EAR), and only 6% of adults consumed more than Chinese Recommended Nutrient Intake (RNI). Chinese adults derived vitamin A mainly from plant source foods, which is supplied as carotenes (67.4% RE or 56.4% RAE). Dark- and light- vegetables and fruits were major contributors of carotenes (accounted for 84.2%). The most import food sources of retinol were egg, meats and meat products, poultry, fish and milk, representing 94.7% of retinol intake. The major four contributors of total vitamin A (as both RE and RAE) were dark vegetables, egg, light vegetables, and meats and meat products. In conclusion, dietary vitamin A remains a problem for Chinese adults.

**Conclusions:**

Public health actions are needed to increase vitamin A intake in China.

## Background

Vitamin A is an essential fat-soluble nutrient for eye health, immune function, embryonic development, cell differentiation and growth hormone production [1]. Low vitamin A intake may lead to blindness and increased morbidity and mortality. Vitamin A deficiency remains a major public-health issue in developing countries, especially in low-income regions, such as south Asia and sub-Saharan Africa [2]. In China, vitamin A deficiency is now considered a moderate public-health problem. On the positive side, recent studies indicate that vitamin A status has been improved in the past decade for Chinese children and pregnant women [3, 4].

Vitamin A is a generic term comprising retinol and provitamin A carotenoids (β-carotene, α-carotene, and β-cryptoxanthin). People obtain dietary vitamin A either as retinol from animal foods or as provitamin A carotenoids from vegetables and fruits [5, 6]. With respect to the rentinol activity, the former equivalent of vitamin A intake is proposed by FAO/WHO (1988) as RE (retinol equivalent) = retinol+(β-carotene/6) + (α-carotene/12) + (β-cryptoxanthin/12) [7],while the present equivalent is introduced by IOM (2001) as RAE (retinol activity equivalent) = retinol + (β-carotene/12) + (α-carotene/24) + (β-cryptoxanthin/24) [1, 8]. Several countries have issued new dietary reference intakes (DRIs) of vitamin A as RAE, such as the United States, Canada, and Australia, where it recommended 900 μg for men and 700 μg for women [1, 9]. Chinese DRIs recommended 800 μg for men and 700 μg for women [10].

It is still unclear whether Chinese adults eat enough to meet the recent recommended dietary intakes of vitamin A as well as its food sources at the national level. In the present article, we examined the dietary vitamin A status (as retinol, carotenoids, RE and RAE) and its food sources among Chinese adults aged 18 to 64 years, using data from the most recent China Nutritional Transition Cohort Study (CNTCS, 2015).

## Methods

### CNTCS and sample

We used data from CNTCS 2015, which was conducted by National Institute for Nutrition and Health, Chinese Center for Disease Control and Prevention. CNTCS was an amplified survey based on the China Health and Nutrition Survey (CHNS), an ongoing and longitudinal study established in 1989 by the Chinese Center for Disease Control and Prevention, and the University of North Carolina at Chapel Hill. Detailed information on the rationale and methods of CHNS can be found on the project website and published papers [11, 12]. CNTCS was designed to provide a wide range of socio-demographic characteristics, diet, health, physical activity, behaviors and environment changes in China. The original sample was collected using a multistage random cluster sampling method from representative households in eight provinces (Liaoning, Shandong, Henan, Hubei, Hunan, Jiangsu, Guizhou and Guangxi) by economic levels, geographical areas, and public resources. During the past decades, more provinces joined the study using the consistent sampling method and protocol: Heilongjiang joined in 1997, three mega cities (Beijing, Shanghai and Chongqing) joined in2011, and three more provinces (Zhejiang, Shanxi and Yunnan) joined in2015.

All subjects gave their written informed consent before they participated in the study. The study was conducted in accordance with the Declaration of Helsinki, and the protocol was approved by the Ethics Committee of the National Institute for Nutrition and Health, China CDC (No.2015017).

The current paper analyzed data from 12,246 adults (5710 men and 6536 women), aged18 to 64 years old, who had complete dietary data on three consecutive days in 2015. The final sample came from 6018 households, residing in 361 communities from15 provinces in China.

### Assessment of dietary carotenes, retinol and vitamin a (RE, RAE) intake

Personal dietary data were collected using 24-h recalls on three consecutive days (two weekdays and one weekend day) by face-to-face interview. The trained interviewers collected detailed information of all foods and beverages consumed at home and away from home in the past 24 h for each individual. Household edible oil and condiments consumption were weighed during the same days and divided among household members according to their dietary energy intake levels.

Dietary carotenes and vitamin A (RE) intake were calculated using the China Food Composition Table 2009 [13]. However, carotenes was tested using chromatography in the China Food Composition Dataset, which could not distinguish the subtypes, such as β-carotene, α-carotene, and β-cryptoxanthin. Other research showed thatβ-caroteneaccounts for 86% of provitamin A carotenoids intake [14]. Therefore, we used the conversion factor of β-carotene instead that of total carotenes in the calculation procedures. Retinol and vitamin A (RAE) were derived from the following simplified formulas:

1 $$ \upmu \mathrm{g}\;\mathrm{RE}=\upmu \mathrm{g}\;\mathrm{retinol}+\upmu \mathrm{g}\;\mathrm{carotenes}/6, $$

2 $$ \upmu \mathrm{g}\;\mathrm{RAE}=\upmu \mathrm{g}\;\mathrm{retinol}+\upmu \mathrm{g}\;\mathrm{carotenes}/12. $$

Food groups were defined according to the classification in China Food Composition Tables. Carotene was the major contributor to vitamin A intake in vegetables, which varied in different vegetable types. Thus, we defined vegetables with carotene more than 500μg/100 g as dark vegetables, which mainly were dark green, red or orange (such as spinach, broccoli, tomato, carrot and pumpkin); and those with carotene less than 500μg/100 g as light vegetables (such as eggplant, cucumber and cabbage).

### Socio-demographic variables

Socio-demographic variables used in the present analysis include: age, gender, education and income level, and regions. Age (years) was categorized to 2 groups, 18–49 years, and 50–64 years. Highest level of education attained was categorized to 3 groups, primary school andbelow, junior middle school and senior middle school and above. Per capita annual household income was classified into 3 groups using tertile method, low (1^st^tertile), middle (2ndtertile), and high (3rdtertile). Ethnic groups were divided into Han, Miao, Buyi, Man, and others. To explore region disparities on vitamin A intake, we included 3 variables, region1 (urban, rural), region2 (east: Being, Liaoning, Shanghai, Jiangsu, Zhejiang and Shandong; middle: Heilongjiang, Henan, Hubei and Hunan; west: Chongqing, Guizhou, Yunnan, Shanxi and Guangxi), and region3 (north: Beijing, Liaoning, Shandong, Heilongjiang, Henan and Shanxi; south: Shanghai, Jiangsu, Zhejiang, Hubei, Hunan, Chongqing, Guizhou, Yunnan and Guangxi).

### Statistical methods

In our analysis, vitamin A was expressed as both retinol equivalent (RE) and retinol activity equivalent (RAE). The present paper reported dietary carotenes, retinol, vitamin A (RE), and vitamin A (RAE) intakes (mean of the three days for each adult) as mean (SD) and median by age, gender, region, education, income and ethnic groups. The intake levels of vitamin A (RAE) were assessed using estimated average requirement (EAR) and recommended nutrient intake (RNI) from Chinese Dietary Reference Intakes (DRIs) 2013 [15]. The proportions of retinol to total RE and RAE, as well as the main food sources contributing to each of the vitamin A components were also determined. Non-parametric statistical methods were used for difference test by each of the stratifications. Significance level was set at *p* < 0.05. All statistical analysis was conducted using SAS 9.4 (SAS Institute Inc., Cary, NC, USA).

## Results

### Dietary intake of vitamin a among Chinese adults

Table 1 reports the dietary intake of carotenes, retinol, and vitamin A (excluding supplements) by age, gender, region, education, income level and ethnic groups among Chinese adults. Mean total dietary intake of vitamin A was 480.9 μg RE/day (male: 499.4μg RE/day; female: 464.7μg RE/day) or 307.2 μg RAE/day (male: 321.3μg RAE/day; female: 294.8μg RAE/day), carotenes intake was 2084.7 μg/day (male: 2137.7 μg/day; female: 2038.4μg/day), and retinol was 133.5 μg/day (male: 143.2μg/day; female: 125.0μg/day).

**Table 1:** Dietary daily intake of Vitamin A among Chinese adults aged 18 to 64 years*

Variables	N	%	Carotenes (μg/day)				Retinol (μg /day)				Vitamin A (μg RE/day)				Vitamin A (μg RAE/day)			
			Mean	SD	Median	*P*	Mean	SD	Median	*P*	Mean	SD	Median	*P*	Mean	SD	Median	*P*
Total	12,246	100.0	2084.7	3061.2	1175.0		133.5	264.4	76.3		480.9	583.5	307.6		307.2	374.3	202.0	
Age																		
18–49 years	6875	56.1	2099.2	3092.5	1175.4	0.7079	141.6	281.3	78.0	< 0.0001	491.4	598.1	311.8	0.0485	316.5	389.9	205.7	0.0179
50–64 years	5371	43.9	2066.0	3021.0	1172.9		123.1	240.6	74.2		467.4	564.1	302.8		295.2	353.0	198.8	
Gender																		
Male	5710	46.6	2137.7	3132.4	1194.7	0.0975	143.2	279.8	81.4	< 0.0001	499.4	600.4	318.5	< 0.0001	321.3	388.9	211.2	< 0.0001
Female	6536	53.4	2038.4	2997.2	1158.8		125.0	249.8	72.3		464.7	567.9	298.0		294.8	360.6	195.5	
Region 1																		
Urban	4781	39.0	2114.9	2985.9	1259.0	0.0001	147.4	268.6	91.2	< 0.0001	499.9	568.0	332.5	< 0.0001	323.6	368.1	224.2	< 0.0001
Rural	7465	61.0	2065.3	3108.6	1129.8		124.5	261.2	67.7		468.7	593.0	290.5		296.6	377.9	190.4	
Region 2^§^																		
East	4659	38.1	1695.4	2827.1	905.4^a^	< 0.0001	149.9	268.4	94.9^a^	< 0.0001	432.4	548.2	277.8^a^	< 0.0001	291.2	361.6	193.1^a^	< 0.0001
Middle	3239	26.5	1667.0	1911.6	1044.2^b^		105.2	183.1	70.6^b^		383.0	372.5	271.4^a^		244.1	246.6	180.6^b^	
West	4348	35.5	2812.9	3780.0	1675.6^c^		136.9	306.2	63.6^c^		605.8	713.1	383.9^b^		371.3	449.3	238.2^c^	
Region 3																		
North	4713	38.5	1357.7	1758.3	801.7	< 0.0001	108.0	191.4	71.9	< 0.0001	334.3	358.1	238.3	< 0.0001	221.1	246.9	162.2	< 0.0001
South	7533	61.5	2539.5	3572.6	1494.2		149.4	300.1	78.7		572.6	671.9	365.3		361.0	426.8	234.2	
Education level^§^																		
Primary school and below	3104	25.4	1906.0	2702.1	1062.3^a^	< 0.0001	115.4	249.4	61.0^a^	< 0.0001	433.1	519.6	274.8^a^	< 0.0001	274.3	339.6	180.7^a^	< 0.0001
Junior middle school	4268	34.9	2123.6	3187.4	1199.2^b^		126.3	256.5	70.7^b^		480.3	605.4	301.7^b^		303.3	381.5	197.3^b^	
Senior middle school and above	4874	39.8	2164.3	3158.3	1226.3^b^		151.2	279.0	91.2^c^		511.9	600.4	333.1^c^		331.5	387.1	225.5^c^	
Income level^§^																		
Low (1^st^tertile)	4081	33.3	1982.9	2913.0	1113.1^a^	0.0001	133.5	297.0	65.0^a^	< 0.0001	464.0	585.2	285.8^a^	< 0.0001	298.8	395.5	188.0^a^	< 0.0001
Middle (2^nd^tertile)	4079	33.3	2191.6	3172.7	1230.1^b^		126.1	244.7	73.3^b^		491.4	589.7	313.2^b^		308.7	365.9	205.2^b^	
High (3^rd^tertile)	4086	33.4	2079.6	3089.4	1198.5^b^		140.8	248.0	90.0^c^		487.3	575.4	317.7^b^		314.0	360.5	214.2^c^	
Ethnic groups^§^																		
Han	11,053	90.3	2078.9	3133.4	1150.0^a^	< 0.0001	134.5	265.1	77.9^a^	< 0.0001	481.0	594.3	305.2^a^	< 0.0001	307.8	378.9	201.0^a^	< 0.0001
Miao	221	1.8	2855.7	2514.2	2400.0^b^		107.9	228.8	57.1^b^		583.8	494.0	471.1^b^		345.8	322.9	281.1^b^	
Buyi	182	1.5	3117.7	2132.7	2683.3^c^		100.5	243.4	51.5^c^		620.1	476.4	503.9^b^		360.3	334.0	277.6^b^	
Man	225	1.8	994.1	1195.6	633.3^d^		125.8	274.0	71.0^a,b^		291.5	339.4	211.3^c^		208.6	291.9	148.4^c^	
Others	565	4.6	1997.3	2347.8	1351.2^a^		136.3	264.4	67.4^a,b^		469.1	482.3	328.0^a^		302.7	336.1	205.4^a^	

*Mean, standard error, and median were calculated for the values of vitamin A. Significant difference by socio-demographic factors was determined using nonparametric median test

^§^If the difference was significant among more than two groups, multiple-comparison was further conducted by Student-Newman-Keuls (SNK). The same characters (a,b,c,d) indicated non-significant difference between the two sub-groups, while the different characters indicated significant difference, *p* < 0.05

Higher intake of carotenes was observed in western areas vs. middle and eastern areas, in rural areas vs. urban area, in southern areas vs. northern areas, in Buyi and Miao minorities VS. other ethnic groups. Lower carotenes intake was found in low subgroup compared with middle-high groups of education and income. No significant differences of carotenes intake were detected by age and gender.

Retinol intake was significantly higher in younger adults compared to older adults, and in males compared to females. Opposite to carotenes intake, the higher intake of retinol was observed in urban areas vs. rural areas, and in eastern areas vs. middle and western areas. Adults in southern areas tended to have significant higher retinol intake than those in northern areas. Similarly, retinol intake was found to increase with education and income level. Buyi minorities had the significant lowest retinol intake. Vitamin A intake (expressed in both RE and RAE) showed coincident trends with that of retinol in all stratifications, except for ethnic groups. The highest vitamin A intake level (both RE and RAE) was found in Han, followed by Miao and Buyi, while the lowest level was observed in Man minorities.

### Proportion below EAR and above RNI among Chinese adults

In Chinese DRIs, the estimated average requirement (EAR) and the recommended nutrient intake (RNI) of vitamin A is set to 560 μg RAE/day and 800 μg RAE/day for males, 480 μg RAE/day and 700 μg RAE/day for females. The proportion of Chinese adults who consumed vitamin A less than EAR, as well as the proportion of adults who consumed vitamin A more than RNI is shown in Table 2. In general, the proportion below EAR was 88.3% in males and 87.0% in females; consumption above RNI was only 6.3% in males and 6.5% in females. Younger adults seemed to have a healthier vitamin A intake level than older adults, with a lower proportion below EAR and a higher proportion above RNI, whereas no remarkable gender difference in proportions was found for each age group.

**Table 2:** Vitamin A intake and proportion below EAR and above RNI among Chinese adults by age and gender

Age		N	Vitamin A (μg RAE/day)		*P*	Below EAR^a^		*P*	Above RNI^b^		*P*
			Mean	Median		N	%		N	%	
18–49 Years	Male	3157	330.1	214.7	0.0008	2764	87.6	0.1744	222	7.0	0.9497
	Female	3718	305.0	198.6		3214	86.4		260	7.0	
50–64 Years	Male	2553	310.4	207.8	0.0003	2276	89.2	0.0946	139	5.4	0.4805
	Female	2818	281.5	191.0		2471	87.7		166	5.9	
Total	Male	5710	321.3	211.2	< 0.0001	5040	88.3	0.0313	361	6.3	0.6599
	Female	6536	294.8	195.5		5685	87.0		426	6.5	

^a^EAR, Estimated Average Requirement, 560μg for male, and 480μg for female; ^b^RNI, Recommended Nutrient Intake, 800μg for male and 700μg for female

### Contribution of retinol to total vitamin a intake

**Fig. 1:**
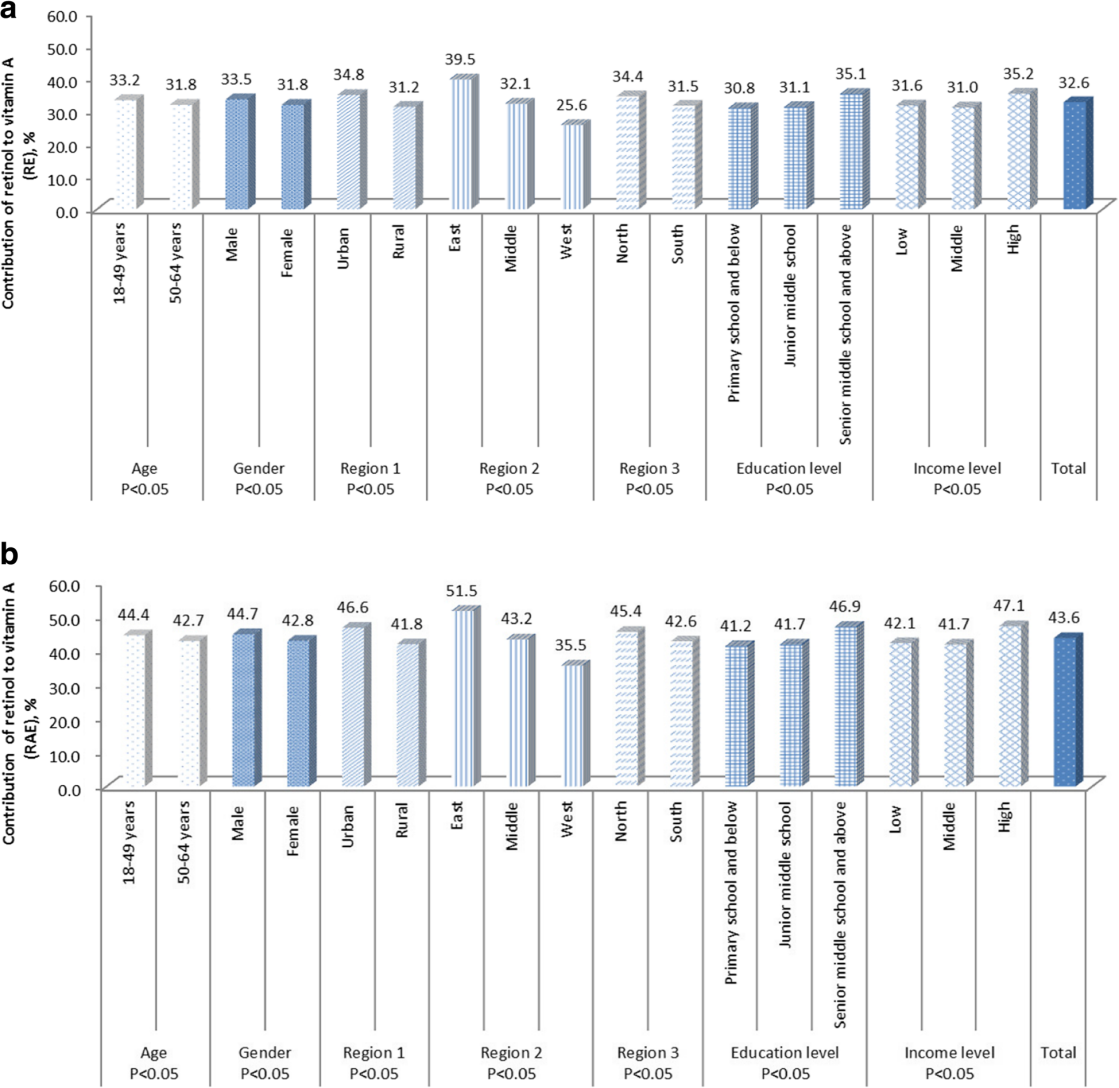
Contribution of retinol to vitamin A among Chinese adults aged 18 to 64 years: (**a**) vitamin A expressed as RE; (**b**) vitamin A expressed as RAE

Figure 1a and b shows the percentage of daily contribution of retinol to total vitamin A intake (RE and RAE). In terms of RE, retinol accounted for 32.6% of total vitamin A intake, which contributed 33.5% in males and 31.8% in females. 43.6% of vitamin A intake was attributed to retinol, when expressed in RAE (44.7% for males and 42.8% for females). Social-economic differences in the contribution of retinol to total vitamin A were detected. Retinol provided a higher proportion of vitamin A in young, male, urban, eastern, northern, higher education, and higher income groups.

### Food sources to the daily vitamin a intake

**Fig. 2:**
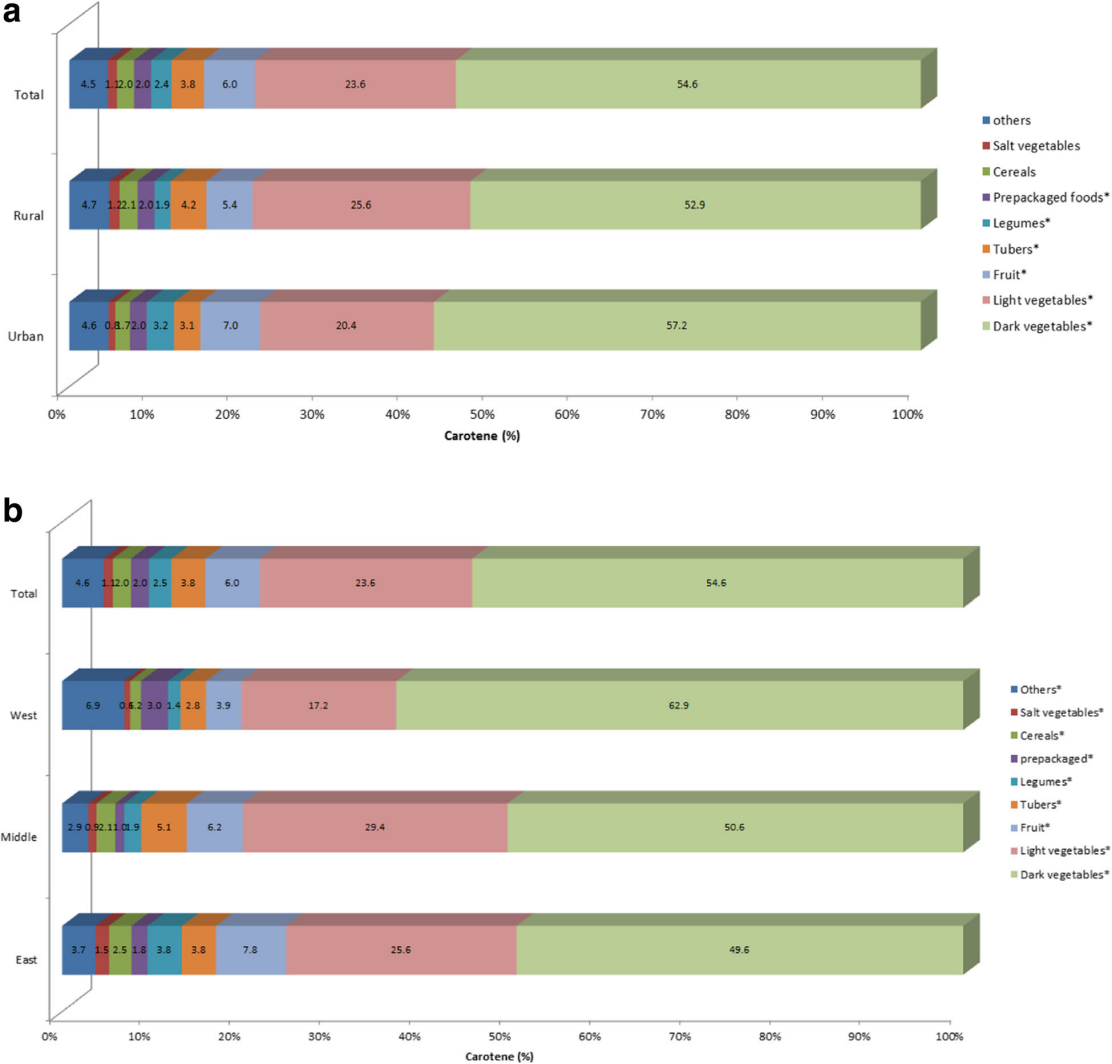
Contribution of food sources to the daily carotene intake by regions among Chinese adults aged 18 to 64 years. *P < 0.05. (**a**) by urban and rural; (**b**) by east, middle and west

Figure 2a shows the percentage contribution of the main food sources to the daily carotene intake in urban and rural adults. Overall, dark vegetables (54.6%) afforded more than half of carotene intake for the whole population, contributing to a higher percentage for urban groups vs. rural group. Light vegetables (23.6%) ranked second and fruit (6.0%) third; tubers contributed 3.8% and legumes 2.4%. Compared with rural adults, urban adults appeared to consume less carotene from light vegetables and tubers, more from fruit and legumes. All the listed food groups in Fig. 2a afforded more than 95% to the carotene intake.

Figure 2b shows the percentage contribution of the main food sources to the daily carotene intake in east, middle and west areas. Compared with adults in middle and east areas, west adults consumed more carotene from dark vegetables, and less from light vegetables, fruit and tubers.

**Fig. 3:**
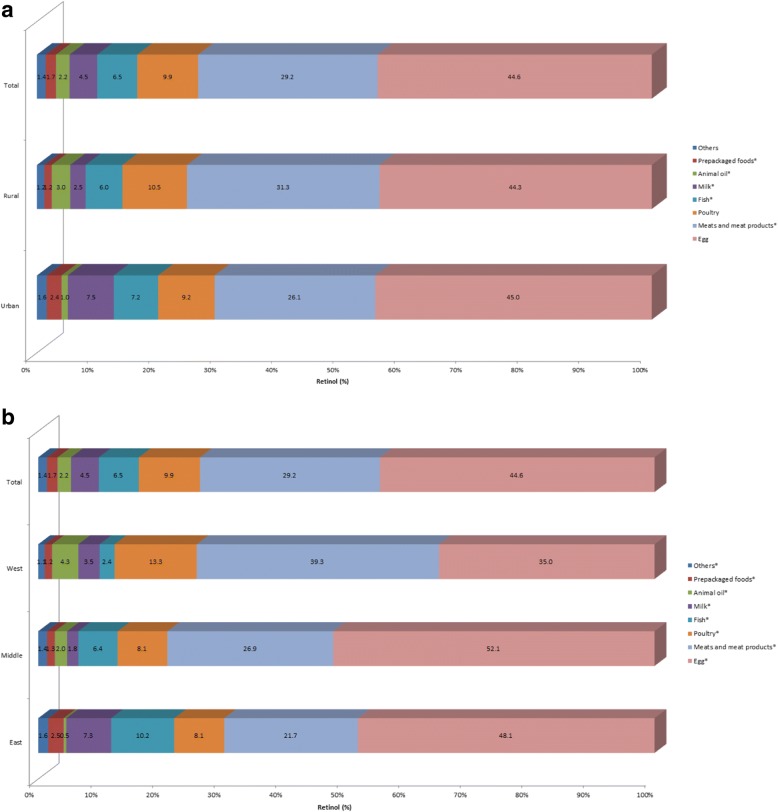
Contribution of food sources to the daily retinol intake by regions among Chinese adults aged 18 to 64 years. *P < 0.05. (**a**) by urban and rural; (**b**) by east, middle and west

As shown in Fig. 3a, eggs provided 44.6% of retinol intake, and were the major contributing food source. Meats and meat products provided 29.2% and poultry 9.9% of retinol intake. Finally, fish and milk afforded 6.5 and 4.5% to retinol intake, respectively. Urban adults seemed to consume more retinol from fish and milk, and less from meats and meat products and poultry than rural adults, with a roughly similar amount from eggs. All the food groups in Fig. 3 combined to provide more than 98% of retinol intake.

Figure 3b shows, unlike middle and east areas, meats and meat products were the major contributing food source in west area, providing 39.3% of retinol intake. Furthermore, west adults seemed to consume more retinol from poultry and animal oil, and less from egg and fish than middle and east adults. Noticeably, fish, milk and prepackaged foods afforded 10.2, 7.3 and 2.5% to retinol intake in east area, respectively, much higher than that in west and middle area.

**Fig. 4:**
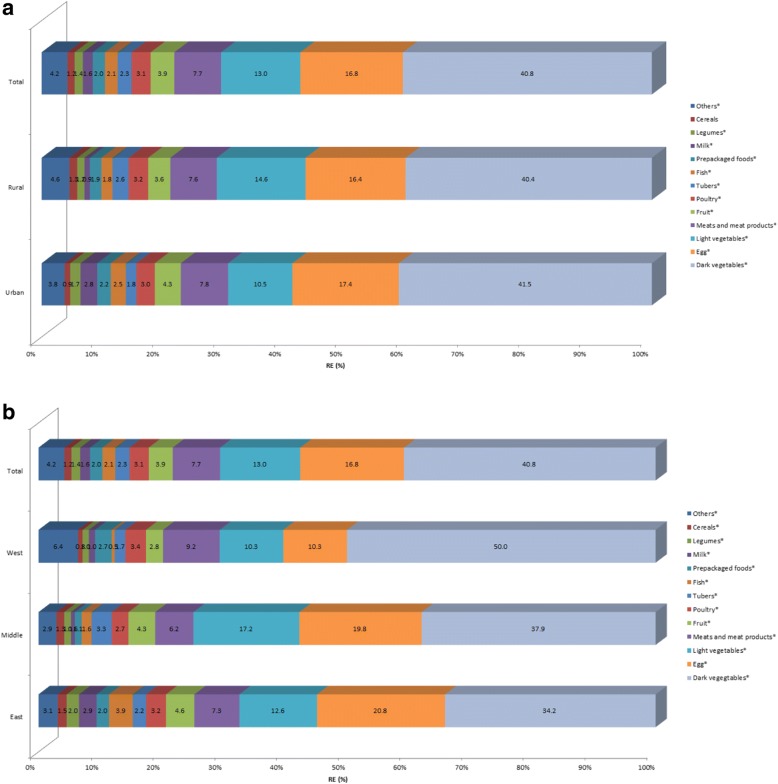
Contribution of food sources to the daily vitamin A (RE) intake by regions among Chinese adults aged 18 to 64 years. *P < 0.05. (**a**) by urban and rural; (**b**) by east, middle and west

**Fig. 5:**
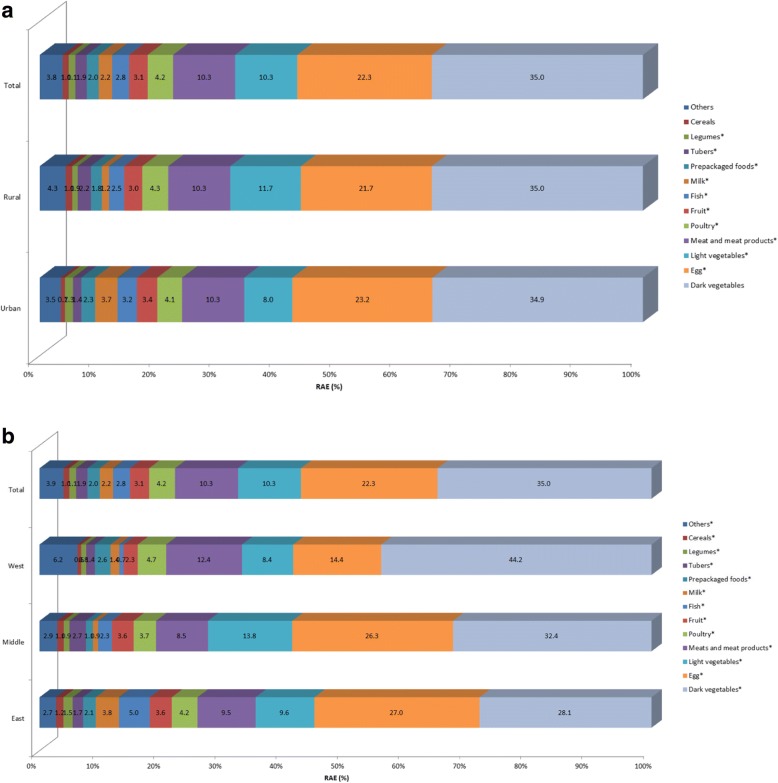
Contribution of food sources to the daily vitamin A (RAE) intake by regions among Chinese adults aged 18 to 64 years. *P < 0.05. (**a**) by urban and rural; (**b**) by east, middle and west

Figures 4 and 5 shows that dark vegetables, eggs, light vegetables, meats and meat products, fruit and poultry are the major food sources ofvitamin A intake, contributing nearly 85% to RE and RAE for the whole population. Due to different conversion factors of carotene in RE and RAE calculation formulas, animal source foods contributed a higher proportion to vitamin A intake for RAE than RE. Urban adults consumed more vitamin A from egg and fruit, while less from light vegetables. Adults in west area consumed more vitamin A from dark vegetables, meats and meat products, and less from egg, light vegetables, fruit and fish, than those in middle and east areas. Interestingly, fish, milk and prepackaged foods combined to provide 8.8% of RE and 10.9% of RAE in east area, which were much higher than that in middle and west areas.

## Discussion

The present article provides a snapshot of dietary intake of vitamin A among Chinese adults. The mean daily intake of vitamin A in this study (480.9 μg RE/day, and 307.2 μg RAE/day) was roughly in line with the findings from Chinese Nutrition and Health Surveillance (2010–2012) (441.9 μg RE/day, and 291.5 μg RAE/day) [16], and that from China National Nutrition and Health Survey 2002 (469.2 μg RE/day) [17]. The above two studies [16, 17] using the nationwide data, expressed the vitamin A intake at standard person level (60 kg adult men with light physical activity), which may overestimate the intake status. Take this into account, our results indicated a mild growth of vitamin A intake among Chinese population during the past decade. Studies in other countries [18–22] usually showed vitamin A intake by gender/age groups for which DRIs have been established. Our research found that dietary vitamin A intake of adults in China was lower than that in Korea (887.8 μg RE/day, and 531.84 μg RAE/day) [18] and Japan (491 to 571 μg RE/day for men, and 603 to 609 μg RE/day for women) [19], and was much lower than that in other European and American countries, such as Italy (890 μg RE/day) [20], Spain (716.4 μg RE/day) [6], the United States(656 μg RAE/day for males, and 564 μg RAE/day for females) [21], and Mexico (547 μg RAE/day for men, and 536 μg RAE/day for women) [22]. When it comes to dietary intake of retinol and carotenes, Chinese adults consumed absolutely less retinol (133.5 μg/day) than those in European countries (835 μg/day), and less carotenes (2084.7μg/day) than most countries, excluding the Netherlands, Sweden, and Spain [5, 23]. Compared to other Asian countries, such as Japan and Korea, Chinese adults had slightly lower retinol intake and much less carotenes intake [18, 19].

Data presented in the current study also shows some very interesting socioeconomic differences in the intake of vitamin A. Higher intake of vitamin A was found in younger, male, urban, western, southern, high education and income groups, and Miao and Buyi minorities. That finding may be explained by the fact that adults in those groups have better access to high quality and balanced foods.

Various countries have released different recommended dietary intake values for vitamin A, either as RE or RAE. These different recommendations lead to varying interpretations on the prevalence of inadequate intake. For example, the Korean vitamin A EARs for males and females (≥20 years) are 500 to 540 μg RE and 430 to 460 μg RE, respectively [24]. The US/Canadian vitamin A EARs are 625 μg RAE and 500 μg RAE for males and females (≥20 years), respectively [1]. The recent Chinese vitamin A EARs are 560 μg RAE and 480 μg RAE for males and females (≥18 years), respectively [15]. The reported inadequate proportions below EAR of vitamin A varied from 9.4 to 70.3% [18, 22, 25]. In our study, 88% of Chinese adults consumed less vitamin A than EAR, and only 6% of adults had higher intake than RNI, which meant an inadequate intake at the population level.

Both retinol and carotenes are the main sources of vitamin A intake. In industrialized, Western countries, retinol accounted for nearly 65% of total vitamin A intake, while carotenes made up only 35% [14]. Unlike the findings from developed countries, the current study indicated that retinol only accounted for 43.6% of total vitamin A intake (RAE) in China. The proportion even declined to 32.6%, when vitamin A intake was expressed as RE. The proportion of retinol to total vitamin A intake also showed a significant socio-economic difference. Adults in young, male, urban, eastern, northern, high education, and high income groups consumed more vitamin A from retinol.

Previous reports showed various food sources of vitamin A in different countries. ANIBES Study demonstrated that, among Spanish individuals, milk and products were the main source of retinol (38.7%), followed by eggs (22.6%) and fish (11.4%); vegetables afforded 52.7% of carotenes intake, and then fruits ranked second (13.5%).For total vitamin A intake, vegetables were the main source (31.3%); milk and products provided 21.7%; then eggs ranked third (11%) and fruits fourth (6.9%) [5]. EPIC Study showed that European adults obtained their retinol mainly from meats and meat products (51.7%); added fats and dairy products took the second and third place (18.5 and 15.6%, respectively); vegetables and fruit provided 67.8 and 7.8% of β-carotene intake [23].

Unlike the Western diet [26], dark vegetables and light vegetables were the major contributors of carotene in China (54.6 and 23.6%, respectively), followed by fruit (6.0%) and tubers (3.8%); egg provided 44.6% of retinol; meat and products afforded 29.2%; followed by poultry (9.9%) and fish (6.5%); milk only provided 4.5%. The current study also showed that when vitamin A was expressed as RE the order of the major contributors to vitamin A intake was dark vegetables, eggs, light vegetables, meats and meat products, fruit, poultry, tubers and fish; the order changed slightly when vitamin A was expressed as RAE, with animal source foods contributing more vitamin A and plant source foods less.

China has undergone dramatic socioeconomic changes in the past decades, as well as changes in food consumption and eating behaviors [27]. Although the consumption of animal source foods increased greatly during the past years [28], Chinese adults obtained vitamin A mainly from plant source foods.

It should be noted that there are some limitations in our study. First, vitamin A intake from supplements was not included in the analysis because of insufficient data of supplement use in CNTCS. However, unlike the common use of dietary supplements in the Western world [29, 30], supplement use was far lower in China [31], which leads to little bias of the results. Second, specific carotenes components, such as α-carotene, β-carotene, and β-cryptoxanthin, were not distinguished in the China food composition table and thus went beyond the scope of this paper.

Other study showed that adequate dairy intake greatly improved vitamin A intake, especially among women and older adults [25]. Meanwhile, in the recent Chinese Dietary Guidance (2016) [32], the Chinese Nutrition Society encourages people to consume plenty of vegetables, milk and soybeans, as well as an appropriate amount of fish, poultry, eggs and lean meant.

## Conclusions

The present study highlights that, according to the current recommended values, there was a substantial percentage of Chinese adults who had low intakes of vitamin A. Compared with adults in developed countries, the intake of vitamin A, especially retinol intake, was much lower among Chinese adults. The findings illustrate the public health actions are needed to tackle the inadequate intake of vitamin A in China.

## Abbreviations

CHNS: China Health and Nutrition Survey
CNTCS: China Nutritional Transition Cohort Study
DRIs: Dietary Reference Intakes
EAR: Estimated Average Requirement
RAE: Retinol Activity Equivalents
RE: Retinol Equivalents
RNI: Recommended Nutrient Intake
